# Iron-Based Water Treatment Residuals: Phase, Physicochemical Characterization, and Textural Properties

**DOI:** 10.3390/ma14143938

**Published:** 2021-07-14

**Authors:** Magdalena Likus, Małgorzata Komorowska-Kaufman, Alina Pruss, Łukasz Zych, Tomasz Bajda

**Affiliations:** 1 Faculty of Geology, Geophysics and Environmental Protection, AGH University of Science and Technology, A. Mickiewicza 30, 30-059 Kraków, Poland; 2 Faculty of Environmental Engineering and Energy, Institute of Environmental Engineering and Building Installations, Poznan University of Technology, Berdychowo 4, 60-965 Poznań, Poland; malgorzata.komorowska-kaufman@put.poznan.pl (M.K.-K.); alina.pruss@put.poznan.pl (A.P.); 3 Faculty of Materials Sciences and Ceramics, AGH University of Science and Technology, A. Mickiewicza 30, 30-059 Kraków, Poland; lzych@agh.edu.pl

**Keywords:** solid characterization, WTRs, water treatment sludges

## Abstract

Groundwater treatment residuals (GWTRs) are safe waste materials generated during drinking water treatment. GWTRs are mainly deposited in landfills, but the preferred solution should be reused or utilized for some components. To ensure proper sludge management, it is important to provide quality, chemical composition, and texture characteristics of GWTRs. Therefore, in this study, we aimed to investigate and compare the features of GWTRs collected from four water treatment plants. GWTRs were characterized by X-ray diffraction (XRD); scanning electron microscopy (SEM) with energy dispersion spectroscopy (EDS); Fourier transform infrared spectroscopy (FTIR); thermogravimetric, differential thermogravimetric, and differential thermal analysis (TG, DTG, and DTA, respectively); X-ray fluorescence (XRF); inductively coupled plasma optical emission spectrometry (ICP-OEP); specific surface area (S_BET_) measurement; and determination of the isoelectric point (pH_IEP_). According to the results, GWTRs are poor crystalline materials that are predominantly composed of ferrihydrite with minor calcite and quartz admixture. They formed heterogeneously mixed particles with irregular shapes. They were mainly composed of iron oxides (32–55%), silica (4–28%), calcium oxide (4–17%), and manganese oxides (0.3–4.0%). They were found to be mesoporous with a large specific surface area. Due to their composition and texture characteristics, GWTRs demonstrate good adsorption properties toward different compounds such as heavy metals and metalloids.

## 1. Introduction

Drinking water treatment residuals (WTRs) are nonhazardous by-products generated during the treatment of drinking water in water treatment plants, and so far, there is no proper application available for GTWRs [1]. The quantity of WTRs usually varies from 2% to 5% of the whole volume of the processed water [2]. In this decade, the production of WTRs has sharply increased, and thousands of tons of WTRs are produced every day, which tends to be deposited [3,4]. Moreover, landfill disposal is increasingly expensive. WTRs may be a potentially usable material but are being wasted as there is no application available so far. From the viewpoint of the “3R principle” (reduce, reuse, and recycle), it is crucial to identify a possible management option for WTRs [5], which might prove to be beneficial both in terms of environmental safety and economy [6,7].

The origin (surface or underground), as well as the physicochemical composition of the water, determines the appropriate type of treatment. For example, surface water is purified using coagulation, flocculation, sedimentation, and filtration methods [8], whereas groundwater is purified by removing iron and manganese compounds [9]. Hence, WTRs can be classified into two categories. First, post-coagulation residuals or surface water treatment residuals (SWTRs) are primarily composed of amorphous masses of iron or aluminum hydroxides due to the presence of Fe and Al salts which are used to remove suspended solids and humic substances from raw water [10]. Second, residuals arising from the treatment of groundwater with a high concentration of Fe(II) and Mn(II) or infiltration water treatment called groundwater treatment residuals (GWTRs). Usually, groundwater treatment is a simple, nonchemical reagent technology based on aeration and filtration processes [11]. The iron salts present in groundwater easily hydrolyze to soluble Fe(II) hydroxide and then oxidize and precipitate as soluble Fe(III) hydroxide, which is the primary component of the sludge. These metals are removed from groundwater to avoid organoleptic problems for consumers and to avoid technical problems in facilities that supply groundwater [12,13]. Moreover, the permissible level of Fe and Mn in drinking water is less [14,15]. To the best of our knowledge, there is less information with respect to the composition, properties, and potential of GTWRs. Therefore, in this study, we aimed to study the phase, physicochemical, and texture characteristics of GTWRs.

The structure of GWTRs is primarily composed of ferrihydrite. Ferrihydrite (Fe_5_OH_8_∙4H_2_O) is poorly crystalline iron oxide. Because of their amorphous nature, GWTRs have a large specific surface area and are highly reactive [16]. Therefore, GWTRs demonstrate a high capacity for adsorbing a large number of contaminants [17]. In addition, iron-containing materials such as red mud, bog iron ore, and drinking WTRs were previously used as sorbents. They seem to be promising as they cause low risk to the environment. However, GWTRs contain heavy metals such as Al, Fe, As, Ba, Be, Cd, Co, Cr, Cu, Mn, Mo, Ni, Pb, and Zn, but their concentration is usually low (except for Fe). The aforementioned heavy metals occur in relatively stable forms, and according to the US Environmental Protection Agency (EPA), WTRs are nonhazardous materials [18,19,20].

In recent years, many researchers are focusing on reusing of GWTRs. An increasing number of articles on the use of GWTRs for the removal of many contaminants reveals that there is a great requirement for a more detailed examination of these materials. Studies have shown high adsorption capacity of GWTRs for cadmium [21], manganese [22], phosphorus [23], arsenic [24], and nickel [25]. A comprehensive study in terms of the mineralogical, physical, and textural parameters of GWTRs is fundamental in the development of a potential application of GWTRs.

Therefore, in this study, we aimed to investigate and compare the features of GWTRs from four different water treatment plants. The phase and chemical composition, as well as textural and physicochemical properties of GWTRs, was evaluated based on scanning electron microscopy equipped with energy dispersive spectrometer (SEM-EDS); X-ray diffraction (XRD); Fourier transform infrared (FTIR) spectroscopy; thermogravimetric (TG), differential thermogravimetric (DTG), and differential thermal analysis (DTA); X-ray fluorescence (XRF); inductively coupled plasma optical emission spectrometry (ICP-OEP); and specific surface area (S_BET_). The pH_IEP_ of samples was also determined. So far, there has been no research that has used so many different methods of analysis to characterize the GWTRs. Researchers have focused only on the basic characteristics, including simple methods such as XRD or SEM-EDS. Therefore, the manuscript presents a set of results that have not been published before.

## 2. Materials and Methods

### 2.1. GWTRs

The samples of GWTRs (GWTRs-1, GWTRs-2, GWTRs-3, GWTRs-4) were respectively collected from four groundwater treatment plants (GWTP-1, GWTP-2, GWTP-3, GWTP-4) located in Wielkopolska Voivodeship, Poland. All of these plants extract groundwater from quaternary deposits from depths ranging from 27 to 83 m. The groundwater is treated with typical nonreagent technology based on the process of aeration, filtration, and disinfection. At GWTP-2, the open aeration system with reaction chamber and rapid filtration with the use of open filters were applied, whereas, at GWTP-1, GWTP-3, and GWTP-4, water was treated in pressurized systems. In all four stations, quartz sand or two-layer anthracite-sand bed (GWTP-2 and 3) was used as filter filling. Filter backwashing waters were discharged to the clarifier. At GWTP-2, polyelectrolyte was added to backwash water to accelerate the sedimentation of suspended solids in the clarifier.

The groundwater samples from GWTPs-1–4 were characterized as follows: iron content ranging from 1.0 to 3.62 mg/L, manganese ranging from 0.130 to 0.382 mg/L, elevated turbidity ranging from 6.4 to 27 Nephelometric turbidity units (NTUs), and watercolor ranging from 10 to 200 mg Pt/L. The hardness of water (caused by the presence of Ca and Mg) ranged from 235 to 352 mg CaCO_3_/L. The content of organic compounds was quite different, as evident from the total organic compound (TOC), which ranged from 1.2 to 4.7 mg C/L. Table 1 shows the characteristics of raw and treated water.

Treated water was characterized by a low concentration of iron ranging from 0.020 to 0.116 mg/L, manganese ranging from 0.005 to 0.025 mg/L, turbidity ranging from 0.20 to 0.54 NTU, and the color of water ranging from 2.5 to 5.0 mg Pt/L. The TOCs ranged from 2.1 to 4.7 mg C/L. Treated water fulfilled the requirements for the quality of water intended for human consumption [15]. 

The presented values of the parameters determining the water quality were provided by the Water Treatment Plants (WTPs) and come from the period preceding the sampling of the GWTR (lasting 3–12 months depending on the WTP). These parameters are determined regularly in order to control the WTP work and ensuring the required quality of purified water supplied to the recipient. All parameters were determined in accredited laboratories in accordance with Polish Standards, which correspond to Standard Methods for Water and Wastewater Treatment guidelines [26].

TOC was determined on a TOC analyzer using the high-temperature combustion method. The determination of the carbon content was made by thermocatalytic decomposition of sample in the presence of an N/C catalyst at 800 °C with synthetic air as the carrier gas. The Total Carbon (TC) and Inorganic Carbon (IC) were measured, and the difference between them was calculated to obtain TOC.

GWTR samples were collected from the bottom of the backwash water clarifier. At GWTPs-1–3, sludge samples were collected directly from the upper sediment layer immediately after the clarified backwashings were discharged from the settling tank. At GWTP-4, the sample was collected during the periodic (once every 6 months) cleaning of the clarifier, and it could be collected from deeper layers.

The samples were dried for 4–7 days at 40 °C, ground in a mortar, and sieved using a 1 mm sieve to remove large particles. Then, the powdered samples were subjected to phase analysis in addition to analyzing the chemical composition and porosity.

### 2.2. Method of Analysis

XRD analysis was conducted on a SmartLab RIGAKU diffractometer (RIGAKU Tokyo, Japan) using a copper X-ray tube, in the angular range of 2–70° 2θ with a 0.05° 2θ measuring step. To identify mineral phases, the PCPDFWIN version 1.30, formalized by JCPDS-ICDD, was used. The phases were identified based on the results using the X-RAYAN computer software (Version 4.2.2, “KOMA”, Warsaw, Poland). The chemical composition was determined with sequential wavelength dispersive X-ray fluorescence (WDXRF) RIGAKU using ZSX Primus II with Rh anode (4.0 kW), and emission spectrometers inductively coupled plasma (Perkin Elmer, Tokyo, Japan). A qualitative spectral analysis was performed by identifying spectral lines and determining their possible coincidences. Based on this, analytical lines were selected. The semiquantitative analysis was developed using the SQX calculation program. Loss of Ignition (LOI) was determined by the mass change of calcined sample at 950 °C within 1 h. The FTIR spectra were recorded by Nicolet 6700 spectrometer (Fishers, Waltham, MA, USA) using the drift technique with 64 scans at 4 cm^−1^ resolution in the 4000–400 cm^−1^ region. The powdered sample was mixed with KBr at 2% by weight relative to KBr. Peak fitting was conducted with OMNIC v8.3 software (Thermo Fisher Scientific, Waltham, MA, USA). Morphological observations of uncoated samples were analysed by SEM-EDS (FEI QUANTA 200, FEI, Graz, Austria). The characteristics of porosity were determined on the basis of low-temperature adsorption and nitrogen desorption isotherms at −196 °C. The analysis was conducted using the ASAP 2020 (Micromeritics, Norcross, GA, USA) apparatus for precise sorption measurements in a wide range of relative pressures, from approximately 10^−3^ to 0.99. Before the measurement, the samples were heated under vacuum at 105 °C for 12 h.

TG/DTA coupled with the measurement of the composition of evolved gases was performed using a Netzsch STA 449 F3 Jupiter apparatus (Netzsch, Chennai, India). Samples were heated at a temperature of 30–100 °C (heating rate: 10 °C/min). The analyses were conducted in combustion conditions. The concentration of trace elements was determined by inductively coupled plasma–mass spectrometry (ICP-MS) ELAN 6100 (Perkin Elmer), except for Ba, Sr, and Zn, which were analyzed by ICP-OES Plasm 40 (Perkin Elmer). The isoelectric point (pH_IEP_) of samples was determined by using Zetasizer Nano-ZS (Malvern Inc., Malvern, UK). For the measurement, 0.05 g of the sample was dispersed into both 100 mL of H_2_O and 100 mL of NaCl solution (0.01 M), and its pH value was adjusted with HCl.

## 3. Results and Discussion

### 3.1. XRD Analysis

**Figure 1:**
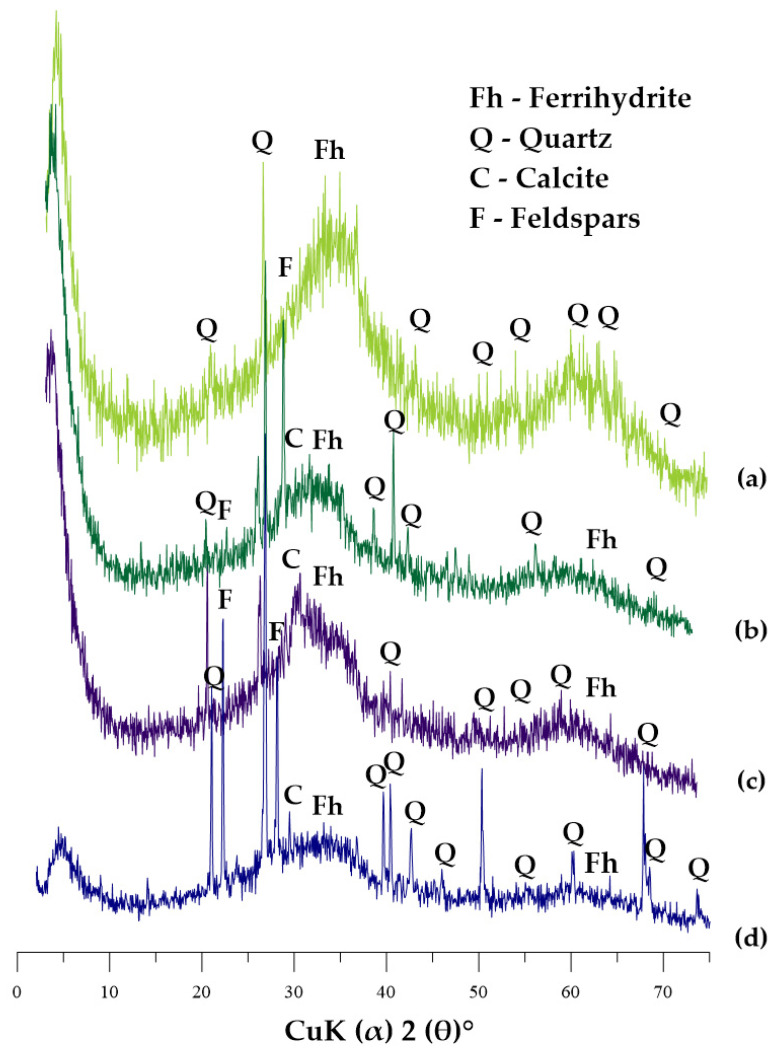
X-ray diffraction patterns of GWTRs. (**a**) GWTRa-1, (**b**) GWTRs-2, (**c**) GWTRs-3, and (**d**) GWTRs-4.

Figure 1 shows the XRD patterns of GWTR-1–4. The XRD patterns did not reveal many sharp peaks, which indicates poorly ordered particles in the GWTRs, and while another method indicates a predominance of iron oxides in GWTRs, there is likely to be amorphous iron [27]. Depending on the reaction conditions, fast and simultaneous oxidation and hydrolysis of Fe(II) salts lead to the formation of lepidocrocite, magnetite, goethite, and poorly crystalline ferrihydrite, as well as feroxyhyte; therefore, the last two can be considered as the most likely components of GWTRs [24,28,29]. Then, 2-line ferrihydrite (Fe_2_O_3_ 5H_2_O) with a very low degree of crystallization can be confirmed on the XRD pattern by two fuzzy diffraction peaks with maxima at about 33–35° 2θ and 61–63° 2θ [30,31]. The rising background pattern of diffractograms indicates the presence of a large number of substances, probably iron compounds, with a low degree of structural order, which agrees with previous research [24,32,33]. All GWTRs contained quartz, which was attributed to the fragments of quartz-containing materials turned out during the backwashing process. GWTR-4 sample (Figure 1d) was the mostly crystalline material; its sharp peaks (20.9, 26.6, 40.0, 42.5, 46.5, 50.2, 60.0, 67.8, 68.2° 2θ) can be attributed to the presence of quartz [34]. A slight peak at maximum at about 30° revealed the presence of calcite admixtures, which can be seen in GWTRs 2–4 (Figure 1b–d). GWTR-2 and 4 contained smaller quantities of feldspar (Figure 1b,d). Manganese compounds could not be identified by XRD analysis, which might be because of the low concentration of manganese, as well as due to its poor structure order [29]. 

### 3.2. Chemical Composition

Table 2 shows the chemical composition of the four GWTRs. It is known that the chemical characteristics of GWTRs depend on the raw water quality and the method used for the treatment (Table 1). Iron removed from water is retained in the form of suspensions in the deposit and washed away during the backwashing process, which is then led to sludges. Manganese grows over the grains and stays predominantly in the filters, and is not washed away. Hence, the concentration of manganese in sludge, as well as treated water, is definitely, which has been proven by XRF analysis [24]. The iron content was the highest in GWTR-1 (56% wt. as Fe_2_O_3_), followed by GWTR-3 (45% wt.), GWTR-4 (37% wt.), and GWTRs-2 (32% wt.). The high iron content in the GWTRs samples is due to the presence of ferrihydrite. The samples showed a clear differentiation in the type of ferrous material, from almost pure form (GWTR-1, 56% wt.) to high content of siliciclastic material (29% wt. SiO_2_, GWTR-4), which is proved by XRD analysis. The backwashing procedure might be responsible for the presence of iron compounds (as in the case of GWTRs-1 and 3), whereas, for GWTRs-4, the quartz peaks are the most visible peaks. The presence of SiO_2_ might have been a result of the residue of quartz sand grains of the filter bed, which was lost during backwashing. Attention should be paid to the dependencies between the concentration of dominant (SiO_2_, Fe_2_O_3_) and secondary components. The GWTRs containing more SiO_2_ are also characterized by large quantities of Al_2_O_3_, Na_2_O, K_2_O, and TiO_2_, probably due to the presence of admixtures of aluminosilicates. Moreover, CaO content is lower in samples rich in Fe_2_O_3_This tendency was not observed for GWTRs-4. The proportion of CaO is the result of natural carbonate precipitation. The loss of ignition (LOI) depended on the quantity of CaO—the higher the concentration of CaO, the higher the LOI. Then, with the increase in the concentration of Fe_2_O_3_, MnO also increases, which is similar to the composition of raw water. The level of MnO in GWTRs is similar to the level of Mn in the treated water (Table 1). The content of P_2_O_5_ did not directly correlate with the content of iron in samples, but the GWTRs-1 that contained the lowest amounts of phosphorus oxides was characterized by the highest amounts of iron oxides. Previous studies have shown that iron oxides can be simply formed by the oxidation of Fe^2+^ in natural water. However, due to the presence of other ions such as silica, calcium, or manganese, as well as organic matter in groundwater, the process of crystallization is constricted [35,36], which is demonstrated by the results of this study. Despite the high content of iron oxides, as demonstrated by XRF analysis, it was an amorphous form of iron compounds. The phase composition of GWTRs, obtained from XRD analysis, showed that the samples are, among others, composed of ferrihydrite (Figure 1), which had the lowest degree of crystallization compared to goethite, hematite, or magnetite. Moreover, the rising background pattern of diffractograms might indicate the presence of amorphous iron compounds. These results can be proved by other research [37,38,39,40,41]. The particle size of ferrihydrite is less than 10 nm [42]. Hence, ferrihydrite, due to its high reactivity and large surface area, was considered a superior absorbent [43].

Trace elements also demonstrated significant differentiation (Table 2). Barium was the leading contaminant in all four samples, varying from 5.46 ppm in GWTR-1 to 15.83 ppm in GWTR-4. The analyzed GWTRs also contained large amounts of arsenic, especially GWTR-4 (4.217 ppm). As the previous research has shown, As is well adsorbed in the beds of quick filters; hence, As might have been adsorbed in iron hydroxides. This can explain the high As content in GWTRs-4 [44,45,46]. There was no significant relationship between the main contents of elements in the samples. GWTR-2–4 contained significant quantities of strontium; the highest concentration was recorded for GWTR-2 (3.91 ppm). This may be because of the large proportion of CaO in the sample, which might be because of the substitution of Sr for Ca. Chromium was present in all four samples in relatively large concentrations, varying from 0.694 ppm (GWTR-2) to 0.990 ppm (GWTR-1). It can be noted that GWTR-4, which has the highest concentration of SiO_2_, also contained the highest quantities of Ba (15.83 ppm), As (4.218 ppm), Zn (2.25 ppm), Sr (2.06 ppm), Cr (0.935 ppm), and Se (0.013 ppm).

The utilization of materials in the environment requires that these materials meet certain environmental regulations. As the GWTRs are waste materials generated from various industrial processes, it is important to assess the leaching behavior of metals and anions. Despite this, concentrations of metals are typically low and similar to those in soils, and most metals and metalloids occur in relatively stable forms; some concern was also associated with the concentration of heavy metals in WTRs [18,19,47]. According to the results from the toxicity characteristic leaching procedure (TCLP) and synthetic precipitation leaching procedure (SPLP) [48], the concentration of trace elements in leachate (As, Ba, Cd, Cr, Pb, Hg, or Se) was below the US EPA-allowable levels and below the US primary or secondary drinking water standards. This suggests that WTR samples cannot be considered hazardous waste. It is noteworthy that so far, no research has demonstrated the toxic effect of WTRs on the environment, which suggests that it can be a safe material for use as an adsorbent.

### 3.3. FTIR Analysis

**Figure 2:**
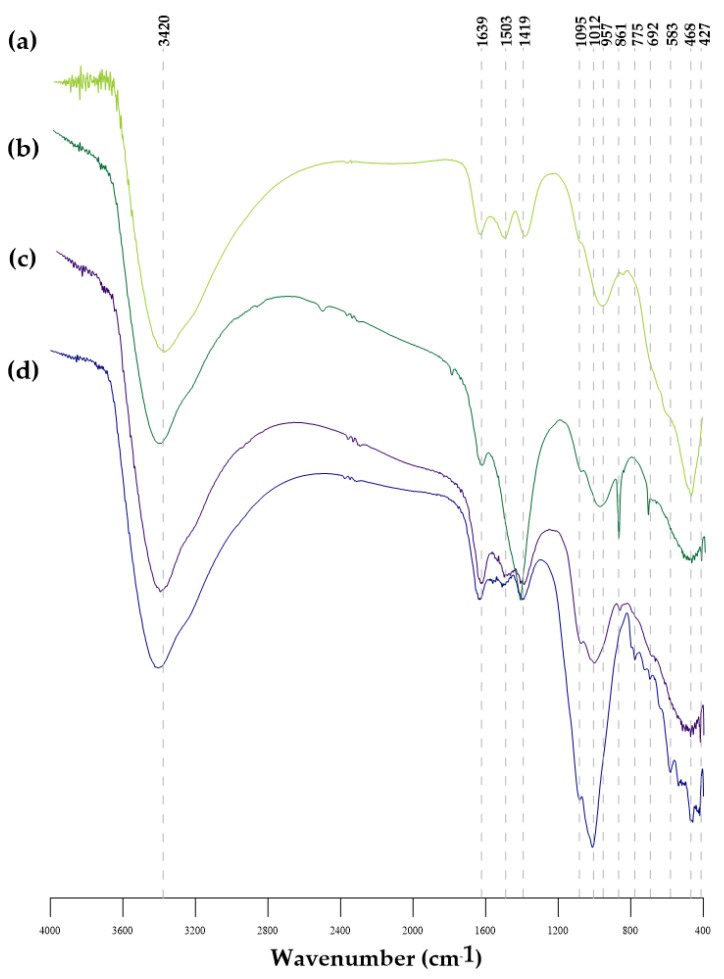
Fourier transform infrared (FTIR) spectra of: (**a**) GWTRa-1, (**b**) GWTRs-2, (**c**) GWTRs-3, (**d**) GWTRs-4.

Figure 2 shows the results of the FTIR analysis of GWTRs-1–4. The FTIR spectra showed that the main peak (3420, 1639, 1419, 1092, 957 cm^−1^) positions and values were similar for all four samples; however, there was a slight variation in the peak positions and intensity of GWTRs-2 and 4. The FTIR spectra of GWTRs-1 and 3 were similar. The broad peaks visible at the 3420 cm^−1^ region were attributed to the characteristic peak of the –OH stretching vibrations of H_2_O [49]. The band located at the 1639 cm^−1^ region was assigned to C=O stretching vibrations [50]. In the case of GWTR-2, the peak appeared at 1419 cm^−1^, which is associated with the symmetrical stretching vibration of O=C-O, whereas for GWTRs-1–3, the stretching vibration was significantly reduced. The aromatic ring with a C=C bond is shown at 1503 cm^−1^ in the case of GWTRs-1, 3, and 4 [51]. The strong band at 1012 cm^−1^ visible in the spectra of GWTRs-3 and 4, as well as the slight shift to wave number 957 cm^−1^ for GWTRs-1 and 2, with a shoulder at 1095 cm^−1^, were the result of the formation of the binuclear complex between ferrihydrite and sulfates [39,52]. Ferrihydrite was characterized by very vague and fizzy absorption bands in the infrared region; therefore, the unambiguous identification of this mineral by FTIR technique was possible only at high concentrations [53].

The FTIR spectra of GWTR-4 (Figure 2d) displayed peaks at 861 cm^−1^ and 775 cm^−1^ that are attributed to Si–O bonds of silica and quartz, which correspond with the XRD and XRF results [54]. The Fe–O stretching vibrations appeared in the 400–600 cm^−1^ area [55]. Moreover, the last sharp peak at the wave number 468 cm^−1^ with a shoulder at 692 cm^−1^ visible for GWTRs-1 and 4 can be attributed to Fe–O and Mn–O stretching vibrations [24,25]. In the case of materials containing mixtures of metal oxides, an accurate consideration is required as in the region of bands with low wavenumbers characteristic for Mn–O vibrations might be observed [56].

### 3.4. TG/DTA 

**Figure 3:**
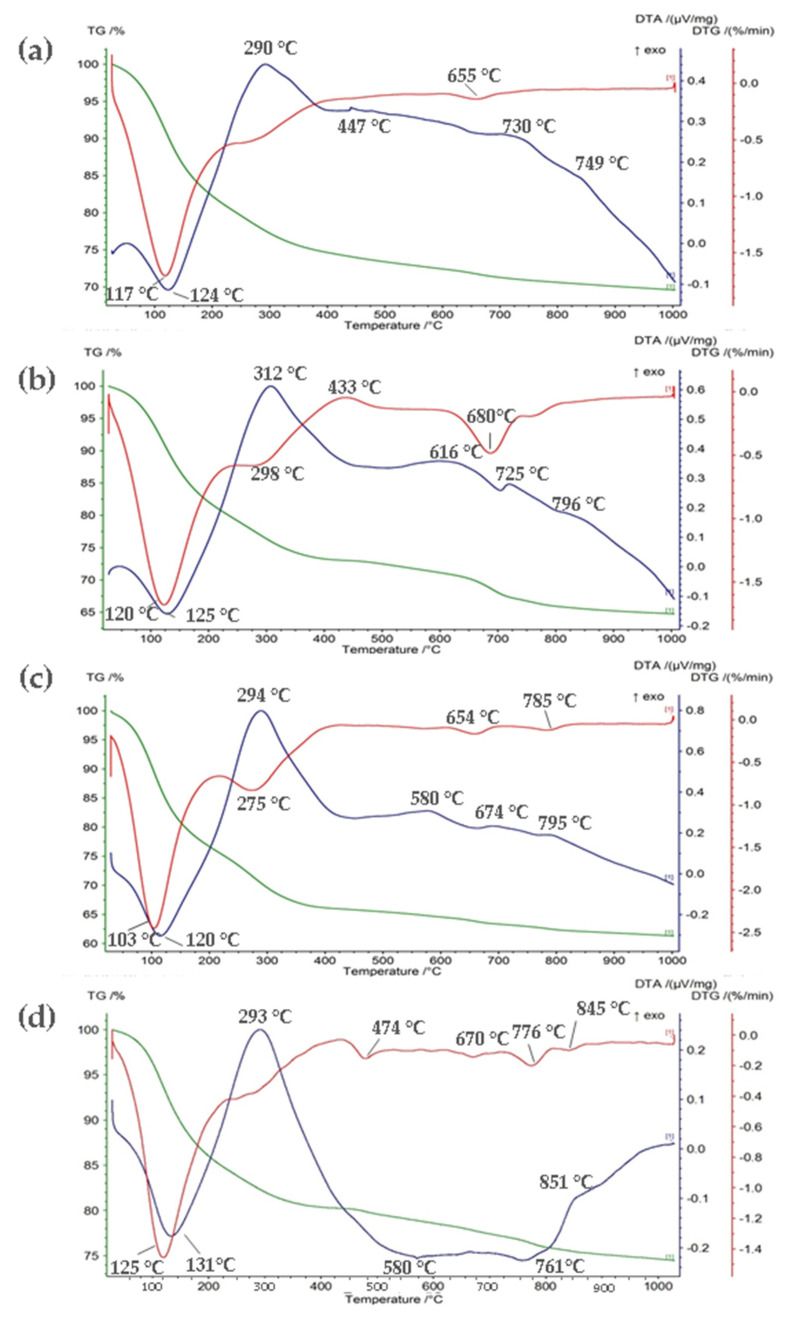
TG, DTG, and DTA analysis of: (**a**) GWTRa-1, (**b**) GWTR-2, (**c**) GWTR-3, (**d**) GWTR-4. The color of the curve corresponds to the color of the Y scale. TG—thermogravimetric, DTG—differential thermogravimetric, DTA—differential thermal analysis.

Figure 3 shows the TG and DTA curves of GWTRs. The course of GWTRs-1–3 thermograms was similar. The DTA curve revealed a very deep endothermic effect in the low-temperature range associated with the removal of weakly bound water [57]. The minimum of this effect is around 120–125 °C. It smoothly turned into an equally intense exothermic effect with a maximum of around 290–312 °C, which can be associated with the decomposition of the organic matter present in the GWTR samples [58]. For GWTR-1, the curve has a slightly different course, and the exothermic effect was not that distinct, which was confirmed by XRF analysis and the highest concentration of iron of all samples. This effect might also be related to the transformation of ferrihydrite into hematite. The exothermic peak is usually relatively sharp [59]. This exothermic peak is indicative of the high energy released during its recrystallization to hematite [60,61]. However, the TG/DTA analysis cannot confirm the presence of ferrihydrite in the case of GWTR samples due to the high content of organic substances [62]. TG analysis showed a 17–23% loss of mass, which is related to the desorption of adsorbed water that takes place at 100–200 °C and the subsequent loss of mass (8–11%) at 200–400 °C. Further loss of mass, usually associated with endothermic effects, occurred at higher temperatures. They were predominantly related to the thermal dissociation of carbonates and the decomposition of residuals of highly transformed organic substances (450–500 °C). At temperatures above 600 °C, the effects resulting from dehydroxylation and breakdown of the structure of GWTRs were mainly observed. The last loss of mass was recorded at higher temperatures up to 900 °C. The very low exothermic effect around 760–795 °C did not cause changes in phase composition and crystal structure. It might be the conversion of the poorly crystalline hematite to a highly crystalline phase [63]. Total loss of mass varied from 29% (GWTR-1) to 39% (GWTR-3).

DTA curves of GWTR-4 indicate the occurrence of several endothermic and exothermic effects (Figure 2d). At temperatures ranging from 130 °C to 290 °C, endothermic and exothermic effects were observed, as in the case of GWTRs-1–3. At temperatures above 580 °C, another deep endothermic effect was noticed, which is probably associated with structural breakdown. TG analysis showed a 14% loss of mass due to the dehydration process at 100–200 °C and the following loss of mass—6% and 4%. This result shows that GWTR-4 had a lower loss in total mass (26%) than that of GWTRs-1–3, which might be related to the high concentration of SiO_2_ and relatively low concentration of Fe_2_O_3_.

### 3.5. BET Analysis

**Figure 4:**
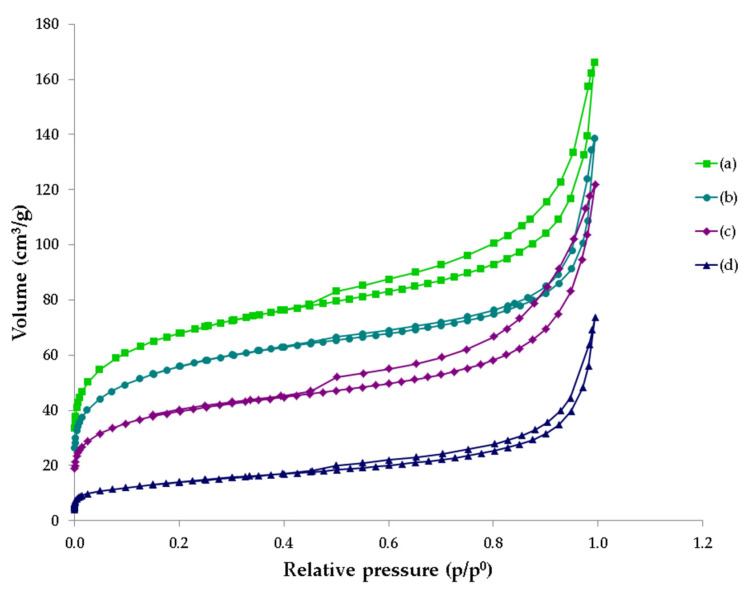
Comparison of N_2_ adsorption and desorption isotherms at −196 °C for (**a**) GWTR-1, (**b**) GWTR-2, (**c**) GWTR-3, (**d**) GWTR-4.

Figure 4 shows the results of the nitrogen adsorption/desorption. The BET analysis revealed that, according to the International Union of Pure and Applied Chemistry (IUPAC) classification, GWTRs-1 and 3 are quite different from GWTRs-2 and 4 [64]. GWTRs-1 and 3 displayed a type II isotherm with a type H3 hysteresis loop (Figure 4). This suits a mesoporous character with the construction of slit-shaped pores originating from the stacking of crystal particles [64,65], whereas GWTRs-2 and 3 had a composite of type IV isotherms with hysteresis loop of type H3, having adsorption curve characteristics of typical mesoporous material [36]. Table 3 shows the results of S_BET_ and porosity analysis. According to the results, the values for S_BET_ were diversified. GWTR-4 showed the lowest value for S_BET_ (49 m^2^/g), whereas GWTR-1 showed the largest one (246 m^2^/g). The large surface area in the case of GWTR-1 is probably because of the highest concentration of iron, as it is well known that iron oxides that existed in the amorphous phase led to its large surface area [29]. GWTRs-2 and 4 had relatively smaller surface areas as they are more crystalline than GWTRs-1 and 3, which was confirmed by XRD analysis. Textural studies have shown the lowest proportion of macropores (15%, 16%, and 22%) in relation to the volume of all pores for GWTR-1, GWTR-3, and GWTR-2, respectively. In the case of GWTR-4, micropores occupied the smallest volume (16%), which is reflected as a relatively small surface area (49 m^2^/g). Mesopores had the largest share of the total pore volume (41–55%). Coagulant-based Fe-SWTRs had a lower surface area (27.5 m^2^/g [66] and 76.8 m^2^/g [67]) than that of GWTRs, which is reported in this study (49–246 m^2^/g), as well as 120 m^2^/g, which was confirmed by Ocinski et al. [24].

### 3.6. SEM Analysis

**Figure 5:**
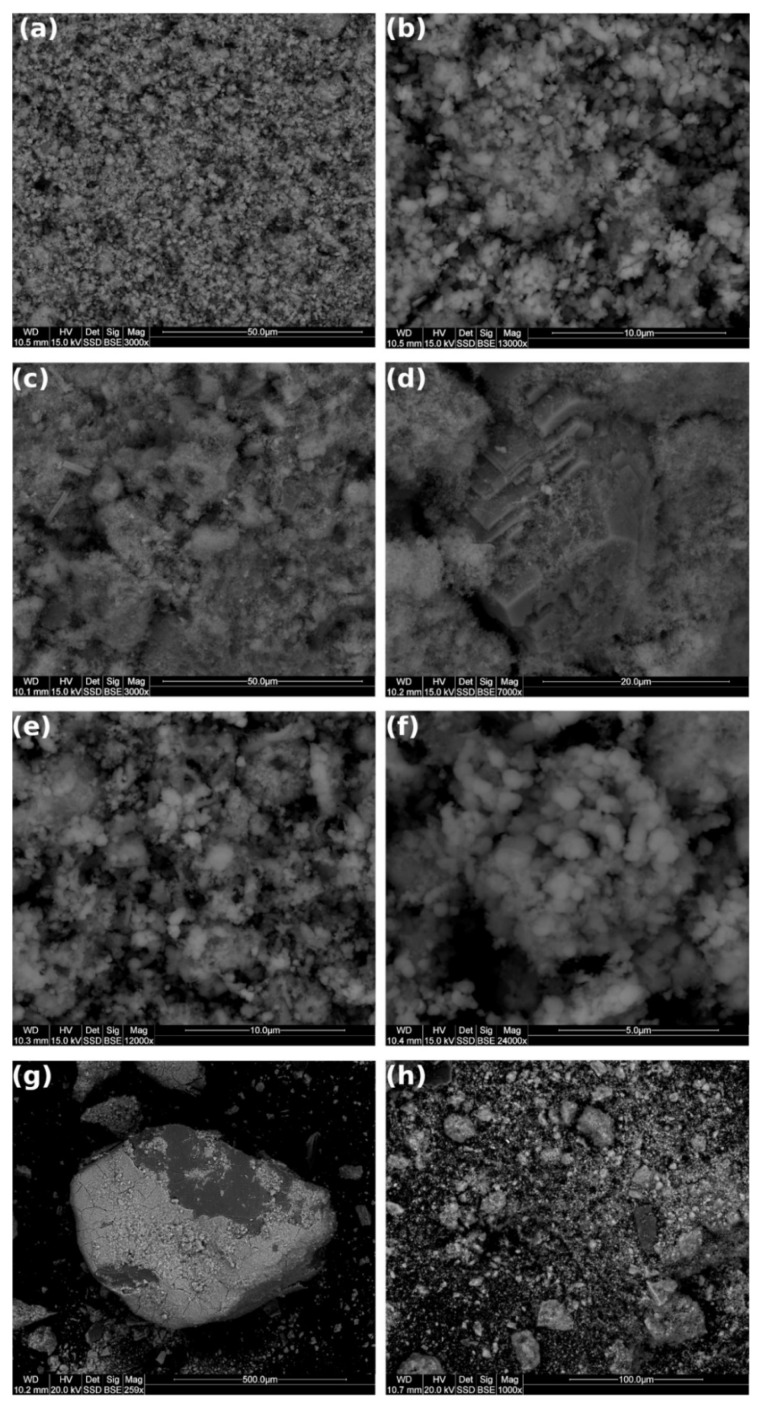
SEM images of samples: (**a**,**b**) GWTR-1; (**c**,**d**) GWTR-2; (**e**,**f**) GWTR-3; (**g**,**h**) GWTR-4 (WD—working distance, HV—high voltage, Det—detector, Sig—signal, Mag—magnification, SSD—Single Shot Detector, BSE—Backscattered Electrons.

The SEM is an effective method to examine surface morphology in the micro-region of environmental samples; therefore, we conducted SEM analysis to study the unique morphological characteristics in GWTR samples [68]. The SEM images (Figure 5) showed a typical microcrystalline-organogenic microstructure. The size of grains varied widely, and the small grains were approximately spherical. It can be observed that the grains were aggregated into large agglomerates. SEM images of all samples revealed that the surface of grains was uneven and rough [48]. Additionally, the surface of the grains showed varying brightness in different regions, which suggests that GWTRs are amorphous [33]. These results also demonstrate the porous nature of GWTRs, which would potentially enhance sorption reactions due to their large surface area [16].

SEM images confirmed the previous results. The amorphous nature of GWTRs was also revealed by SEM images (Figure 5). Figure 5a shows a typical SEM image of GWTRs rich in iron content [3,24]. Differences in shapes and sizes of particles may be observed, and the size of grains varied widely. Figure 5b shows a closer image of GWTR-1 (13,000× magnification); it shows the grains aggregated into large agglomerates, which is the size of several micrometers [24]. Figure 5c,d display small carbonate crystals embedded within substantial cryptocrystalline-aggregated iron oxyhydroxides, which corresponded well with XRD and XRF analysis. Figure 4d represented a fairly smooth surface of the calcite crystal. Magnification of 3000× (Figure 5c) indicated many different types of particles, including needle-shaped fragments, which might be organic composites [69]. Figure 5e,f are similar to Figure 5a,b. The samples had a lot of small particles, which exhibited spherical ball-shaped morphology forming a rough surface with a porous structure [43]. The well-crystalline phase was found to be absent. The residuals were rich in dense iron material, which agreed with the results of the XRD and XRF analysis. The SEM image for GWTR-4 revealed contrasting information when compared with other residuals (Figure 5g,h). The big quartz grain, covered by iron, was visible. The surface of the crystal appeared smooth with several cracks at 259× magnification (Figure 5g), with a rough and porous surface apparent at 1000× magnification (Figure 5h). The particles had irregular surfaces with edges (Figure 5h).

### 3.7. Isoelectric Point

The pH_IEP_ was one of the most important parameters of adsorbents. Table 4 shows the pH_IEP_ of the GWTR samples. According to the results, the pH_IEP_ values ranged from 4.0 to 4.5, and there was a minor difference between the four GWTR samples, which might be caused by different concentrations of functional groups on the surface of the particles that are responsible for its charge [70]. Below this pH, the surface of GWTRs had a positive charge, which favors the uptake of anionic species [70]. However, above this pH, the GWTRs had a negative surface charge, which favors the sorption of cations. This observation was in agreement with a previous study, which reported that the pH_IEP_ was observed at 5 [71].

## 4. Conclusions

In summary, in this study, we investigated the structural and textural characterization of GWTR, which is a product of the de-ironing and de-manganization process of groundwater. Samples were obtained from four different water treatment plants located in Poland. The difference between GWTRs were caused by the chemical composition of raw water, as well as a water treatment technology. According to our results, GWTRs demonstrated amorphous character based on various analyses. The noncrystalline structure increases the surface area of GWTRsN_2_ adsorption/desorption analysis proved the mesoporous structure of GWTRs. The GWTR particles showed irregular shape and size and tended to form aggregates that are a few micrometers in size. The contribution of toxic heavy metals was relatively low, which is considered as an advantage of GWTRs intended to be used in sorption processes. According to our results, GWTRs had good physicochemical characterization and textural properties comparable with commercially available sorbents from the group of zeolites and bentonites. The concerns of increased WTRs production rate and ways of reducing it deserve constant attention. Hence, it is important to develop a novel and feasible solution for the treatment and disposal of GWTRs. Taking into account all presented results, GWTRs should be considered as promising low-cost and effective adsorbents for different pollutants, such as heavy metals and metalloids. Further studies will examine sorption capacity, sorption mechanism, and desorption processes.

## Figures and Tables

**Table 1:** Characteristics of raw and treated water.

Parameters	Unit	GWTP-1		GWTP-2		GWTP-3		GWTP-4	
		Raw	Treated	Raw	Treated	Raw	Treated	Raw	Treated
Color	mg Pt/L	10.0	5.0	33.0	2.5	10.0	5.0	200.0	5.0
Turbidity	NTU	27.0	0.5	15.3	0.5	6.4	0.2	12.4	0.5
Hardness	mg CaCO_3_/L	352	352	290	290	NA *	NA	235	229
pH	—	7.0	7.4	7.3	7.5	7.3	7.2	7.3	7.5
Fe	mg Fe/L	2.490	0.044	3.620	0.020	1.000	0.060	2.900	0.116
Mn	mg Mn/L	0.382	0.007	<0.150	<0.005	0.130	0.004	0.130	<0.025
TOC	mg C/L	2.62	2.10	3.53	3.09	1.20	NA	4.70	4.70

* NA, not analyzed.

**Table 2:** Chemical composition of GWTRs.

Element	Unit	GWTR-1	GWTR-2	GWTR-3	GWTR-4
SiO_2_	%	8.09	4.11	6.15	28.68
TiO_2_	%	0.03	0.02	0.03	0.05
MnO	%	4.33	0.29	2.29	1.36
Al_2_O_3_	%	0.28	0.58	0.60	2.20
Fe_2_O_3_	%	55.76	32.17	44.88	36.51
CaO	%	4.11	17.72	5.85	3.97
MgO	%	0.10	0.37	0.28	0.10
BaO	%	0.13	0.16	0.27	b.d.
K_2_O	%	0.06	0.27	0.12	0.49
Na_2_O	%	0.04	b.d.	0.13	0.51
SO_3_	%	0.22	0.41	0.02	0.07
SrO	%	0.01	b.d.	0.03	b.d.
P_2_O_5_	%	1.69	3.28	8.69	4.85
As_2_O_3_	%	0.03	b.d.	0.03	b.d.
LOI	%	25.12	40.62	30.63	21.21
Se	ppm	b.d.	b.d.	b.d.	0.013
As	ppm	0.710	0.997	0.869	4.217
Zn	ppm	1.86	0.17	0.89	2.25
Sr	ppm	0.50	3.91	1.09	2.06
Ba	ppm	5.46	6.60	10.84	15.83
Cu	ppm	0.341	0.040	0.216	0.092
Ni	ppm	b.d.	0.084	b.d.	b.d.
Cd	ppm	0.021	0.040	0.060	0.041
Cr	ppm	0.990	0.694	0.783	0.935
Hg	ppm	b.d.	b.d.	b.d.	b.d.
V	ppm	0.261	0.310	0.310	0.254

**Notes:** LOI—measured at 950 °C; b.d.—below detection limit.

**Table 3:** Textural parameters of GWTRs.

Parameters	GWTR-1	GWTR-2	GWTR-3	GWTR-4
S_BET_ (m^2^/g)	246	203	144	49
V_tot_^0.99^ (cm^3^/g)	0.249	0.202	0.181	0.105
V_mic_^T^ (cm^3^/g)	0.091	0.073	0.053	0.017
V_mic_^T^/V_tot_^0.99^	0.365	0.361	0.293	0.162
V_mes_ (cm^3^/g)	0.121	0.084	0.098	0.058
V_mes_^BJH^/V_tot_^0.99^	0.486	0.416	0.541	0.552
V_mac_ (cm^3^/g)	0.037	0.045	0.03	0.030
V_mac_^BJH^/V_tot_^0.99^	0.149	0.223	0.166	0.286

Notes: The parameters were calculated as follows: S_BET_—Brunauer–Emmett–Teller methodology; The total pore volume V_tot_^0.99^ for the relative pressure P/P_0_ = 0.99; The volume of micropores V_mic_^DR^—Dubinin–Radushkevich method; The volume of mesopores V_mes_^BJH^—Barrett–Joyner–Halenda (BJH) methodology.

**Table 4:** Determination of the pH of the zero point of charge for GWTRs.

Parameter	GWTR-1	GWTR-2	GWTR-3	GWTR-4
pH_IEP_	4.5	4.4	4.0	4.1

## Data Availability

Data sharing is not applicable.
